# Machine learning-driven imaging data for early prediction of lung toxicity in breast cancer radiotherapy

**DOI:** 10.1038/s41598-025-02617-4

**Published:** 2025-05-27

**Authors:** Tamás Ungvári, Döme Szabó, András Győrfi, Zsófia Dankovics, Balázs Kiss, Judit Olajos, Károly Tőkési

**Affiliations:** 1 Markusovszky University Teaching Hospital, Markusovszky str. 5, Szombathely, 9700 Hungary; 2 Miskolci SZC Bláthy Ottó Villamosipari Technikum, Soltész Nagy Kálmán str 7, Miskolc, 3527 Hungary; 3 Jósa András University Teaching Hospital, Szent István str. 68, Nyíregyháza, 4400 Hungary; 4 University of Nyíregyháza, Sóstói str.31/B, Nyíregyháza, 4400 Hungary; 5 HUN-REN Institute for Nuclear Research, Bem square 18/c, Debrecen, 4026 Hungary

**Keywords:** Artificial intelligence, Breast cancer, Radiotherapy, Machine learning, Lung injury, CT image, Radiotherapy, Computational biophysics, Outcomes research, Breast cancer, Predictive markers

## Abstract

One possible adverse effect of breast irradiation is the development of pulmonary fibrosis. The aim of this study was to determine whether planning CT scans can predict which patients are more likely to develop lung lesions after treatment. A retrospective analysis of 242 patient records was performed using different machine learning models. These models showed a remarkable correlation between the occurrence of fibrosis and the hounsfield units of lungs in CT data. Three different classification methods (Tree, Kernel-based, k-Nearest Neighbors) showed predictive values above 60%. The human predictive factor (HPF), a mathematical predictive model, further strengthened the association between lung hounsfield unit (HU) metrics and radiation-induced lung injury (RILI). These approaches optimize radiation treatment plans to preserve lung health. Machine learning models and HPF can also provide effective diagnostic and therapeutic support for other diseases.

## Introduction

Despite notable advances in therapeutic strategies that have considerably reduced mortality rates, breast cancer remains the most prevalent malignancy among women. According to the National Cancer Institute’s SEER program, the five-year survival rate now exceeds 90%, emphasizing the need to mitigate treatment-related side effects to maintain patients’ quality of life^1,2^. Radiotherapy is a fundamental element of breast cancer management, with most patients undergoing this treatment.

Radiotherapy planning relies on non-contrast computed tomography (CT) images, which provide indispensable data for dose calculations, including tissue density. The Hounsfield Unit (HU) is a fundamental parameter in CT interpretation, quantifying tissue density based on X-ray attenuation. HU values are calculated through a linear transformation of attenuation coefficients, with distilled water defined as 0 HU, air as -1000 HU, and denser materials like bone exhibiting higher positive values^3,4^.

Chest radiotherapy can induce interstitial lung damage. Radiation-induced lung injury (RILI) is a dose-limiting toxicity in thoracic radiotherapy, characterized by a progressive sequence of inflammatory, damaging, and fibrotic responses in lung tissue^5^. Ionizing radiation affects both alveolar epithelial cells and the pulmonary vasculature, leading to the release of pro-inflammatory cytokines and the subsequent activation of fibroblasts and other mesenchymal cells. These changes drive extracellular matrix remodeling and excessive collagen deposition, culminating in radiation-induced fibrosis. The risk and severity of RILI depend on various clinical and dosimetric factors, including total radiation dose, irradiated lung volume, pre-existing pulmonary conditions, smoking history, and overall patient health. The mechanisms contributing to RILI involve inflammation and fibrosis through processes such as alveolar damage, reactive oxygen species (ROS) toxicity, and immune-mediated damage^6–14^. Pulmonary fibrosis can significantly impair lung function, resulting in chronic breathlessness, reduced physical activity, and long-term deterioration in quality of life.

The application of artificial intelligence (AI) and deep learning to medical imaging, particularly in the diagnosis of lung disease, has seen significant advances in recent years. Zarei et al. (2024) performed quantitative analysis on native chest CT images to accurately measure lung lesions, aiding diagnosis and treatment monitoring^15^.

In the area of deep learning and medical imaging integration, Huang et al. (2020) developed a comprehensive system that allows the application of deep learning to combine electronic medical records and imaging to improve diagnosis and treatment processes^16^. Albers et al. (2023) applied high-resolution propagation-based lung imaging at clinically relevant X-ray dose levels, enabling the study of the fine structure of the lung with low radiation exposure^17^. In addition,

examining the reliability of CT texture analysis, Adelsmayr et al. (2023) concluded that 3D segmentation and the use of Hounsfield unit thresholds increased the accuracy of texture analysis in the diagnosis of lung lesions^18^.

These technological advances and artificial intelligence-based applications will contribute to a faster and more accurate diagnosis of lung diseases, as well as personalisation of patient care and treatment, helping to improve treatment efficiency and increase patient survival.

Advanced machine learning techniques, including convolutional neural networks (CNNs) and texture-based classification methods, have significantly enhanced the characterization of lung diseases^19–29^.

In this work the pulmonary fibrosis in breast cancer radiotherapy was investigated, focusing on HU of lungs metrics (minimum, maximum, mean, and standard deviation) and lung volume changes. Three machine learning models and a human predictive factor (HPF) were utilized to predict radiation-induced lung injury (RILI). The HPF is a score derived from Hounsfield unit and lung volume measurements, representing an individual’s risk of fibrosis through a simple method.

A retrospective analysis of 242 patients was conducted to evaluate these parameters and their implications for treatment-related side effects. Among the 242 patients, 113 (46.7%) exhibited detectable radiation-induced lung damage, while 129 (53.3%) showed no radiologically visible lung damage. The study further categorized patients based on the presence or absence of visible lesions, distinguishing between those with lesions and those without^30,31^.

## Results

To investigate the association between CT parameters, lung volume and RILI, three machine learning models (Fine Tree, Kernel-based and k-Nearest Neighbours [kNN]) were used together with a human-generated predictive factor (HPF). The lung volumes (Vcm^3^) of patients, along with the minimum and maximum, mean, standard- deviation of the Hounsfield unit of the affected side lung with and without RILI, were assessed using planning CT scans. The data employed for the construction of the models and the subsequent statistical evaluation are presented in tabular form in (Table 1). Additional comparative data on lung volume and HPF are included in (Table 2).

**Table 1:** Summary of Hounsfield unit (HU) parameters, including minimum and maximum mean values, standard deviation (SD), and lung volumes, among patients with and without radiation-induced lung injury (RILI). The mean, SD, minimum, and maximum values are presented.

Parameter	RILI status	Mean	Std. Dev	Median	Min	Max	*p*-value	Effect size (*r*)
HU minimum	No	−986.39	23.48	−990	−1043	-882	0.123	0.10
	Yes	−978.62	29.71	−988	−1020	-866		
HU maximum	No	242.96	152.25	229	29	840	0.123	0.25
	Yes	266.32	176.29	247	47	1275		
HU mean	No	−746.11	64.13	−749.11	−869.28	−430.38	0.001	0.23
	Yes	−716.03	78.18	−714.68	−846.76	−140.36		
HU STD	No	142.78	18.65	136.45	116.66	192.08	0.001	0.21
	Yes	148.95	17.63	146.47	114.81	195.43		

The Mann-Whitney U-test indicated that the mean HU parameter was significantly higher in patients who developed RILI (median = −714.68) compared to those who did not (median = -749.11), *p* = 0.001, *r* = 0.23 (medium effect). Additionally, the HU standard deviation (SD) scores were higher in the RILI group (median = 146.47) than in the non-RILI group (median = 136.45), *p* = 0.001, *r* = 0.21 (medium effect). However, no statistically significant difference was observed in HU min and HU max values (*p* = 0.123).

**Table 2:** Lung volume (cm^3^) and human predictive factor (HPF) lung volume and human predictive factor (HPF) values in patients with and without radiation-induced lung injury (RILI). Mean, standard deviation (SD), median, minimum, and maximum values are reported. Statistical significance was assessed using the Mann–Whitney U-test. Patients with RILI had significantly lower lung volumes and HPF values, indicating a potential link between these parameters and radiation-induced fibrosis risk.

Parameter	RILI status	Mean	Std. Dev	Median	Min	Max	*p*-value	Effect size (*r*)
Lung volume (V cm^3^)	No	1680.30	450.75	1667.1	693.6	2964.8	0.003	0.19
	Yes	1532.47	466.57	1453.97	668.1	2794.3		
HPF	No	0.14	0.07	0.13	0.03	0.35	< 0.001	0.25
	Yes	0.11	0.05	0.10	0.	0.3		

Lung volumes were significantly lower in the RILI group (median = 1453.97 cm³) compared to the control group (median = 1667.1 cm^2^), *p* = 0.003, *r* = 0.19 (small effect). Additionally, human predictive factor (HPF) values were lower in patients with fibrosis (median = 0.10 vs. 0.13), with a statistically significant difference, *p* < 0.001, *r* = 0.25 (medium effect).

< 0.001, *r* = 0.25 (medium effect).

The HPF is a predictive factor that estimates the probability of developing pulmonary fibrosis based on a number of variables (see Eq. (1)).

The algorithms were trained on 159 samples and validated on 83 samples using five-fold cross-validation. The human-generated mathematical predictive factor (HPF) model displays a slight reduction in accuracy relative to the AI models. Conversely, the straightforward formula facilitates greater ease of use.

**Fig. 1:**
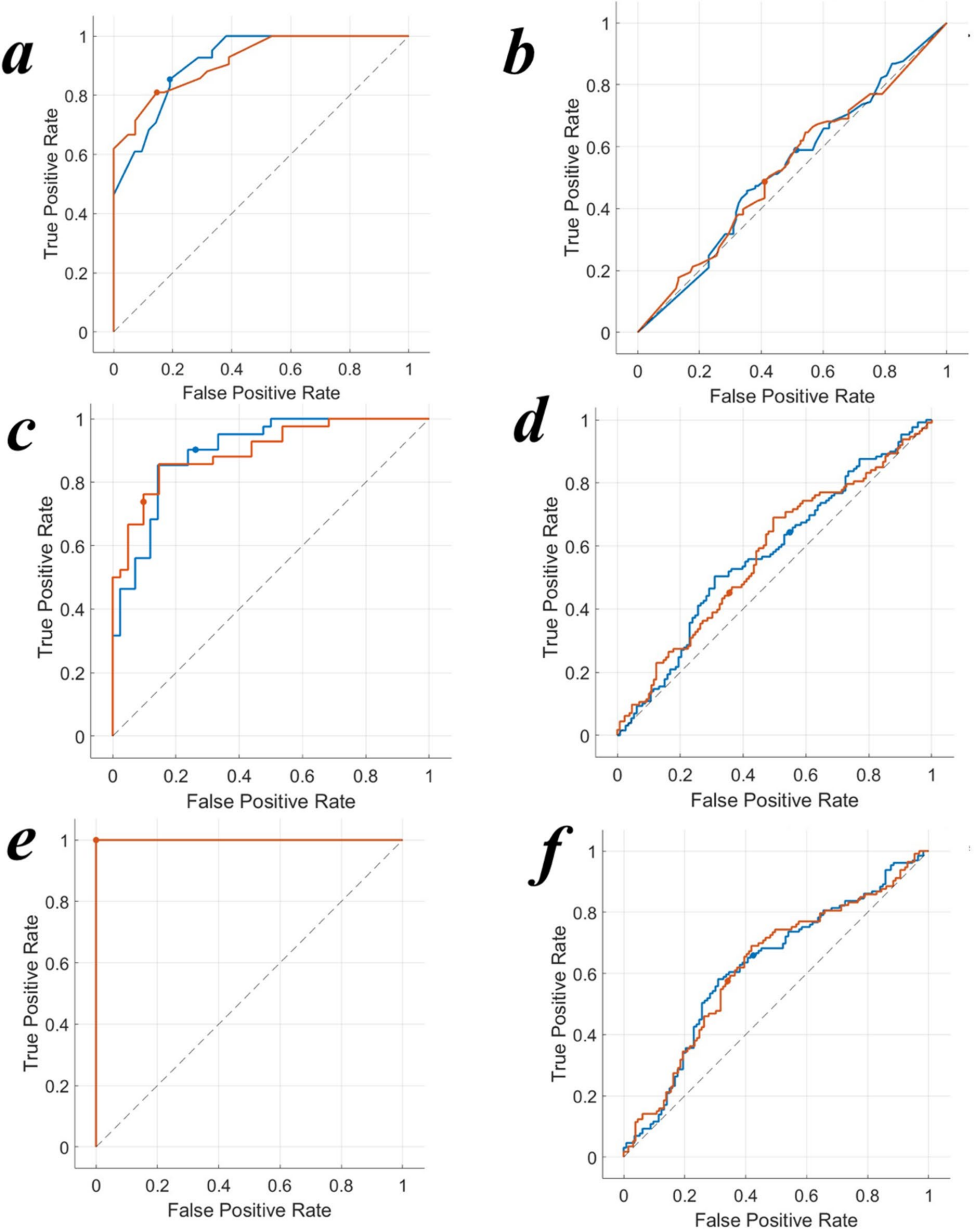
Receiver operating characteristic (ROC) curves for fine tree, test (**a**), and validation (**b**), Kernel-based, test (**c**), validation (**d**), k-Nearest neighbors (kNN) models test (**e**), validation (f). Curves represent test and validation performance in fibrosis prediction.

**Fig. 2:**
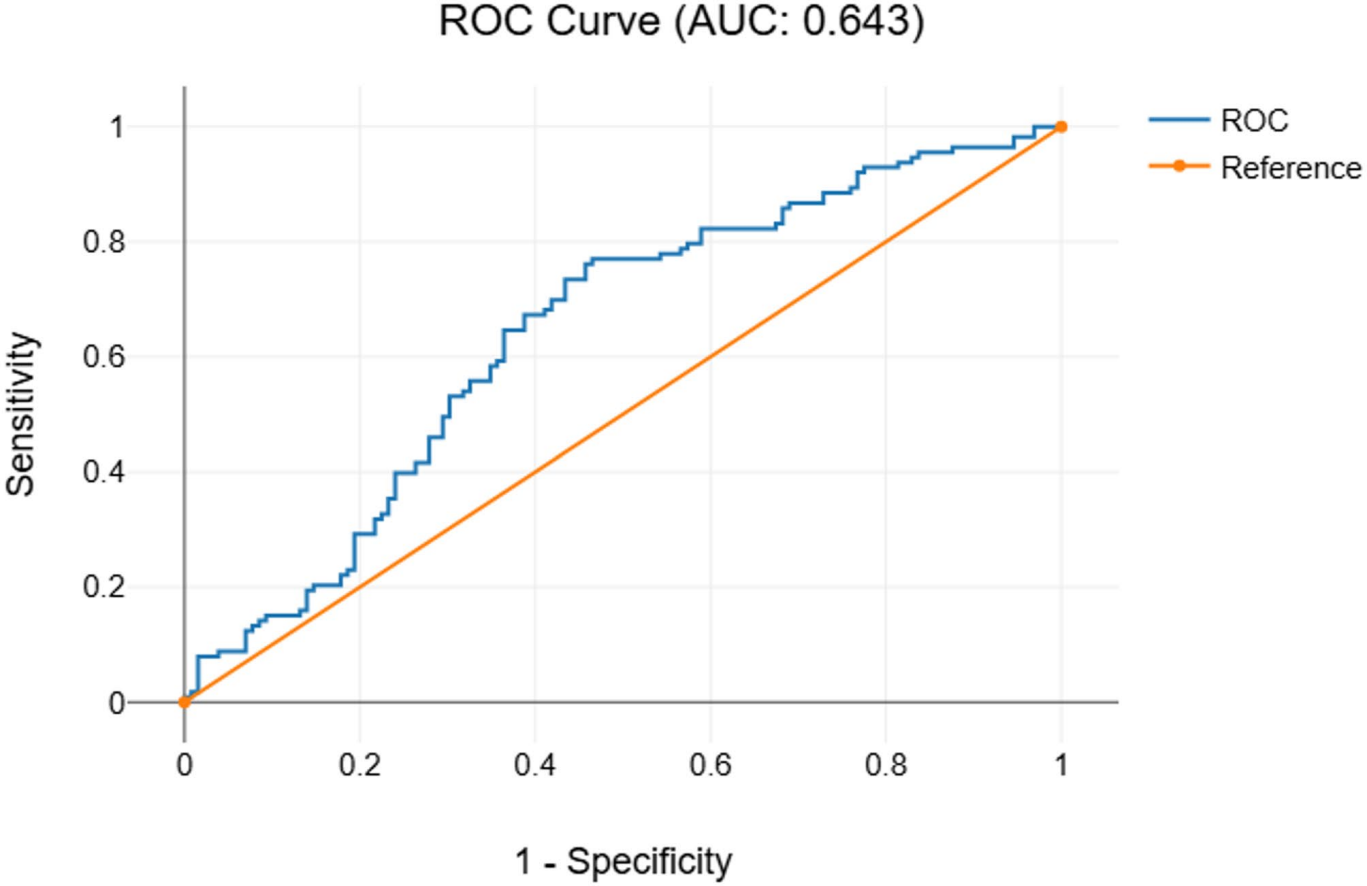
ROC curve for the human predictive factor (HPF) model. The curve demonstrates the trade-off between sensitivity and specificity in fibrosis prediction.

Table 3 provides an overview of the accuracy of the models, while Fig. 1 illustrates the test and validation of the models and Fig. 2 shows ROC curves of HPF.

**Table 3:** Validation and test accuracy of machine learning models and the human predictive factor (HPF). The accuracy values reflect the models’ ability to predict fibrosis risk based on CT-derived parameters and lung volumes.

Model	Accuracy valid %	Accuracy test %
Tree	54.1	83.1
Kernel	55.4	81.9
kNN	62	100
HPF	62.81	72

## Discussion

The application of diverse machine learning algorithms in the medical domain has witnessed a surge in popularity^32–36^.

The statistical analyses, driven by advanced AI models, provided robust evidence of a correlation between RILI and CT-based lung volume parameters. This relationship was further supported by the Human Predictive Factor (HPF), demonstrating that variations in lung Hounsfield Unit (HU) values are strongly associated with fibrosis development.

The significant correlation between lung volume and HU mean (e.g. HU > −720 for fibrosis risk) suggests that these parameters may reflect not only the presence of fibrosis but also the overall health of the lungs. This finding may encourage wider use of radiological data in the prediction of other lung diseases^15,37–39^. In their study, O’Callaghan and colleagues showed that chest CT correlates with the function and radiological features of idiopathic pulmonary fibrosis (IPF) and may serve as a potential biomarker for assessing the severity of IPF disease. Our results on radiation-induced lung injury agree with the above study, where a similar correlation was observed. In order to analyse the radiation-induced changes in Hounsfield Units, Wuschner and colleagues demonstrated in their study that radiation three months after RT causes changes in lung anatomy that exhibit a strong linear correlation with dose. The observed changes in Hounsfield units in the vascular lung parenchyma suggest that this measure may be a potential biomarker of changes in perfusion^40^.

The present study did not investigate the effect of dose on lung tissue. Instead, it focused on lung densitometry based on HU value and lung volume to determine whether this is a biomarker. By comparing the results, it can be confirmed that there may be significant changes in lung HU values before and after radiation treatment. The radiogenic lesion of the lung depends on a number of factors, including the dose received, the lung condition, and the ability to regenerate. Further development of the models is possible, as are tests of other models, for example, the model described by Kadeethum et al.^41^. It is conceivable that the reconstruction and imaging protocols employed in CT scans may influence the HU values of deep learning models, which could subsequently impact the output values.

The ***Fine Tree*** model demonstrated a high test accuracy of 83.1%, highlighting its potential in accurately predicting fibrosis risk. However, the validation accuracy was significantly lower at 54.1%, indicating limited generalizability due to overfitting. The model’s strength lies in its interpretability, providing clear decision rules such as: *“If HU mean > x value*,* fibrosis is likely to develop.”* This rule-based structure may aid clinicians in making rapid, data-driven decisions, though the model’s over-learning of the training dataset presents challenges for broader clinical applicability.

***Optimal Kernel’s*** deep learning model represents a flexible and robust approach, predominantly utilised for classification tasks.

The Kernel-based model achieved a test accuracy of 81.9% and a validation accuracy of 55.4%. Although the validation performance is modest, the model’s capacity to capture non-linear relationships renders it particularly well-suited to complex datasets. This feature is advantageous when analysing the intricate interplay between HU values, lung volume, and patient-specific factors such as age. While the model’s flexibility is advantageous, its sensitivity to outliers underscores the necessity for robust preprocessing in clinical implementations.

The ***kNN*** model demonstrated optimal test accuracy (100%), reflecting its strength in adapting to the training data. However, this exceptional performance underscores a significant limitation: overfitting. The model struggles to generalize to unseen data due to its reliance on proximity-based classification. Nonetheless, its simplicity and efficiency make it a valuable tool for small, well-curated datasets.

A comparative analysis of the ***HPF model*** reveals that it exhibits superior clinical performance due to its straightforward interpretation and expeditious application. Although its accuracy is inferior to that of machine learning models, with testing accuracy at 72% and validation accuracy at 62.81%, the straightforward formulaic approach reduces the potential for overly complex decision-making processes. A high HPF value mean 0.14 (±0.13) indicates a healthier lung structure with a reduced risk of fibrosis. A low HPF mean 0.11(±0.05) indicates the presence of damaged tissue and an elevated risk of fibrosis.

The advantage of the HPF model is that it can be incorporated as an input variable in machine learning algorithms, which can increase their prediction accuracy. This combined approach can increase the efficiency of decision support systems over time.

Unfortunately, its simplicity makes it less accurate than machine kernel or kNN model.

On the other hand, the results of the analysis showed a statistically significant difference in HPF scores between groups with and without fibrosis.

While automated prediction cannot be considered a replacement for medical decision-making, the results can assist doctors in making more rapid and objective diagnostic and therapeutic decisions. The Table 4 presents the test and validation accuracies, as well as an overview of the key advantages and limitations of each model.

**Table 4:** A comparative analysis of the performance of machine learning models and the human predictive factor (HPF).

Model	Validation accuracy (%)	Test accuracy (%)	Advantage	Disadvantage
*Fine Tree*	54.1	83.1	Easy to interpret decision rules	Over-learning, limited generalisability
*Kernel-based*	55.4	81,9	Handling non-linear relationships	Sensitive to outliers
*kNN*	62	100	Simplicity, efficiency on small data sets	Significant over-learning
*HPF*	62.81	72	Simple, fast to implement	Lower accuracy than machine learning models

### Clinical utility

The findings of this study substantiate the correlation between fibrosis and HU parameters, as well as the clinical significance of lung volume. CT parameters may serve as potential biomarkers for predicting lung injury.

The results of the study are as follows: The predictive models (both automated and manual) facilitate the expeditious identification of fibrosis risk. This is of particular importance when developing treatment strategies.

In regard to therapy decision-making, the all model can be employed to optimise radiation treatment plans with the objective of preserving lung health. This may entail the prioritisation of the deep inspiration-breath-holding (DIBH) technique in cases where there is a high risk of adverse effects.

Our models based on human patients yielded comparable results to those observed in Drayson’s study on mice. While the accuracy of our models was inferior, both studies demonstrated the potential of radiomics in identifying radiation-induced lung injury and predicting therapeutic efficacy at early time points^42^. While the accuracy of the models on the current dataset is promising, the small sample size and lack of population diversity limit the generalisability of the results to other patient populations. Although the integration of artificial intelligence in medical imaging has expanded rapidly, the specific application of deep learning to predict radiation-induced lung injury (RILI) remains underexplored. Our study focuses on planning CT data and utilizes both machine learning and a human-derived predictive factor (HPF) to contribute to the early identification of patients at risk of fibrosis, providing a foundation for future, larger-scale investigations.

Further research is required, including a larger and more diverse patient population, to increase the validity of the models. Additionally, attention should be directed towards the radiation dose to the lungs and other factors that can further refine and enhance the models.

## Methods

### Population

The study population comprised breast cancer patients with stage I-III invasive adenocarcinoma or carcinoma in situ who underwent radiotherapy for breast cancer between April 2021 and December 2023^43^. The study included 242 patients for whom a chest CT scan or chest X-ray was available. We did not exclude anyone from the study. The primary outcome was the presence of a shadow (opacity) or bundle (reticulation) in the lung, as observed on chest CT or X-ray imaging, resulting from irradiation.

### Data collection

The data pertaining to the patients, their treatment plans, the follow-up information and the details of the endpoints for pulmonary fibrosis were obtained from the patient registry of the Department of Radiation Oncology at Markusovszky University Teaching Hospital in Szombathely, Hungary. The methodology was approved by the Regional and Institutional Research Ethics Committee of the Markusovszky University Teaching Hospital of Szombathely on 19 September 2022, under the protocol number 26/2022. All experimental procedures were conducted in accordance with the recommendations set forth by the ICH-GCP guidelines. We hereby confirm that the subjects provided informed consent for the appropriate analysis to be conducted. The mean follow-up time was 13.5 months (range: 6–24 months).

### Data definitions and statistics

The radiological images from chest CT scans and chest X-rays were assessed for the presence of lung fibrosis after breast irradiation. The Mann-Whitney U test was used to assess statistical differences between groups with and without RILI for various CT parameters, including HU mean, standard deviation, and lung volume. MATLAB’s built-in cross-validation methods were applied to mitigate overfitting. Statistical analyses were performed using JASP (v, 0.19.0) (JASP 2024 Amsterdam, The Netherlands) and DATAtab: Online Statistics Calculator. ( DATAtab e.U. Graz, Austria.)

### Machine learning models

In this study, three machine learning models—Fine Tree, Kernel-based, and k-Nearest Neighbors (kNN)—were employed to analyze the correlation between CT-derived parameters and the risk of radiation-induced lung injury (RILI). The models were implemented using MATLAB v. R2024b, Classification Learner tool (The Mathworks Inc Natick, Ma US) was used for modelling with a five-fold cross-validation approach to validate their performance. Matlab’s built-in Cross Validation method is recommended for small sample sizes.

### Tree model

A Fine Tree model employs a recursive process of data division into smaller groups based on potential features, with the objective of achieving maximum homogeneity within each final group (leaf). The Fine Tree model, which is based on the decision tree algorithm and is a form of machine learning, is used to address a range of classification and regression issues. This model represents a sophisticated variant of the decision tree paradigm, facilitating the construction of more intricate and comprehensive decision-making structures through the incorporation of multiple branches.

In regression problems, the methodology is analogous, albeit with the final leaves providing a numerical prediction. As the tree becomes more complex and contains a greater number of nodes, it is able to discern subtle differences in the data, and the decision rules are logical and well-interpreted. It is important to note, however, that deep trees are susceptible to overfitting the learning dataset, which can result in a reduction in the model’s generalisability to new data. This is an intrinsic challenge associated with overlearning. Furthermore, the Fine Tree model necessitates greater computational resources due to its enhanced complexity^44^.

Principle: Fine Tree is a decision tree-based model that progressively classifies data according to target variables (e.g., fibrosis present/absent). For this study, the Fine Tree model was configured with a maximum of 100 splits to partition the data. The split criterion used was Gini’s diversity index, and surrogate decision splits were turned off.

### Kernel-based model

This model is an optimizable kernel-based learning technique integrated into a machine learning framework, capable of being combined with features from deep learning architectures^45^. The optimizable kernel is designed to create a unique, problem-specific kernel function that enhances the model’s performance on the given dataset. By using a mathematical function to transform the data into a higher-dimensional space, this approach enables the handling of complex non-linear relationships. In MATLAB, kernel parameters are automatically optimized during the learning process, reducing the need for manual tuning.

While not a traditional deep neural network, this model effectively combines the flexibility of kernel methods with the strengths of deep learning techniques. It is often employed for feature learning, where features extracted from a deep learning network are processed by a kernel-based algorithm. Optimization procedures further improve the model by identifying the best hyperparameters, eliminating the need for extensive manual experimentation. The kernel-based approach is particularly effective for small to medium-sized datasets and has demonstrated superior performance in these cases. This model has been selected for its suitability in analyzing biological and medical data.

For this study, the model was configured with the following hyperparameters: multiclass coding was set to “One vs One,” and the iteration limit was fixed at 1000. During optimization, logistic regression was chosen as the learner, with 115 expansion dimensions, a regularization strength (Lambda) of 0.55035, and a kernel scale ranging from 0.001 to 1000. Data standardization was enabled. The hyperparameter search explored a range of learners, including SVM and logistic regression. The number of expansion dimensions was varied from 100 to 10,000, while the regularization strength (Lambda) ranged from 4.1322 × 10^− 6^ to 4.1322. The standardized data was true and false.

### k-nearest neighbors (kNN) model

The k-Nearest neighbours (kNN) algorithm is a fundamental non-parametric machine learning method widely used for classification and regression tasks. This model was chosen due to its straightforward implementation, as it does not require explicit model training. The algorithm is highly adaptable to various types of data and dimensions, making it a versatile choice in machine learning applications^46^. However, filtering out outliers during preprocessing could negatively influence the results. Despite this potential limitation, the algorithm retains its adaptability and effectiveness.

In this study, the kNN model was configured with 84 neighbors, using the Euclidean distance metric and squared inverse distance weighting. During hyperparameter optimization, the number of neighbors was varied between 1 and 121, and several distance metrics were explored, including City Block, Chebyshev, Correlation, Cosine, Euclidean, Hamming, Jaccard, Mahalanobis, Minkowski (cubic), and Spearman. Additionally, the impact of standardizing the data was evaluated by testing both standardized and non-standardized versions.

### Human predictive factor

HPF is a predictive factor that estimates the likelihood of developing pulmonary fibrosis based on several variables:

1 $$\:\:HPF=\frac{{V}^{\frac{HU\:mean+HU\:sd}{\:HU\:mean-HU\:sd}}}{\:HU\:max-HU\:min}$$.

Lung volume (V in cm^3^) indicates the degree of exertion, with a larger volume associated with a reduced relative risk of tissue damage.

The term “HU mean” refers to the average Hounsfield units of a given lung, which provides an indication of the overall density of lung tissue. A reduction in the mean HU value suggests a healthier lung structure. The HU standard deviation (sd) represents the variability of Hounsfield units in the lung, with higher values indicating greater tissue heterogeneity and potentially the presence of more fibrotic lesions.

The HU maximum and minimum values represent the upper and lower limits of the Hounsfield unit range for the lung.

### Formula interpretation

The following formula should be interpreted as follows:

The numerator represents the lung volume, calculated by dividing the ratio of the Hounsfield unit values. This indicates the degree of risk associated with the condition and volume of the tissue in question.

The denominator is defined as the difference between the maximum and minimum HU values, serving as a normalization factor that accounts for the upper and lower limits of the standard deviation of the HU values.

The most extreme values within the HU range indicate the densest and least dense areas of lung tissue. A broader range of HUs suggests greater tissue diversity.

A predictor is constructed as a function of five variables. Based on the findings from the statistical analysis and prior experience, the objective is to quantify the conditions under which lesions may develop.

### Radiation technique and dosimetry

The breast irradiation was performed in all patients with 3D conformal RT using a CT-based design with mixed energy of 6 and 10 MV or 6 and 18 MV, respectively. The irradiation plan composed of tangential fields and additional beams to optimize the coverage of the target volume of the design and to minimize the dose to the organs at risk: the heart, lungs and contralateral breast.

We note that hypo fractionated total breast irradiation can be used as an equivalent tool to standard radiotherapy for women who have undergone breast conservation surgery for invasive breast cancer with a clear surgical margin and negative axillary nodes^47,48^.

Determining the design target volume and the volume of the “organ at risk” is a critical part of radiotherapy. Identifying volumes on the planning CT is often not an easy task. In our work we followed the European Society for Radiotherapy and Oncology (ESTRO) recommendations^47^.

For the whole breast, a dose of 40.05 Gy^48–51^ was prescribed in 15 fractions (223 cases 92.4%), 50 Gy^46,52,53^ in 25 fractions (17 cases 7.02%), and in one case 43.2 Gy in 24 fractions, depending on pathological risk factors.

The supraclavicular lymphatic area and boost dosimetry for the tumor bed were omitted because tangential field placement was used and did not contribute significantly to the lung burden. Lung contours were generated using Siemens SOMATOM Go.Sim (Siemens Erlangen Germany) CT simulator software (Syngo CT VA40). The image slice width was 3 mm, in accordance with the thorax protocol.

### Toxicity assessment

Subsequent imaging was conducted following the conclusion of the radiotherapy course. The patient presented the radiological results for the consultation. In light of the radiographic findings, the toxicity was assessed as fibrosis, as described by the radiologist, or the presence of reticulation or opacity in the lung at the chest wall, where the irradiation field may have been. All cases were graded as 1 according to the Common Terminology Criteria for Adverse Events v.5. No medical intervention was required, and only radiological lesions were visible.

## Data Availability

The datasets generated and/or analysed during the current study are available in the [https://github.com] repository, [https://github.com/gyorfiandras/Ungvary.git].The underlying Matlab file [and training/validation datasets] for this study is available in https://github.com and can be accessed via this link [https://github.com/gyorfiandras/Ungvary.git].
